# A comparison of altered white matter microstructure in youth born with congenital heart disease or born preterm

**DOI:** 10.3389/fneur.2023.1167026

**Published:** 2023-05-12

**Authors:** Kaitlyn Easson, May Khairy, Charles V. Rohlicek, Christine Saint-Martin, Guillaume Gilbert, Kim-Anh Nguyen, Thuy Mai Luu, Élise Couture, Anne-Monique Nuyt, Pia Wintermark, Sean C. L. Deoni, Maxime Descoteaux, Marie Brossard-Racine

**Affiliations:** ^1^Advances in Brain and Child Development (ABCD) Research Laboratory, Research Institute of the McGill University Health Centre, Montreal, QC, Canada; ^2^Department of Neurology and Neurosurgery, Faculty of Medicine and Health Sciences, McGill University, Montreal, QC, Canada; ^3^Division of Neonatology, Department of Pediatrics, Montreal Children’s Hospital, Montreal, QC, Canada; ^4^Division of Cardiology, Department of Pediatrics, Montreal Children’s Hospital, Montreal, QC, Canada; ^5^Department of Medical Imaging, Division of Pediatric Radiology, Montreal Children’s Hospital, Montreal, QC, Canada; ^6^MR Clinical Science, Philips Healthcare, Mississauga, ON, Canada; ^7^Division of Neonatology, Department of Pediatrics, Jewish General Hospital, Montreal, QC, Canada; ^8^Department of Pediatrics, Centre Hospitalier Universitaire Sainte-Justine, Montreal, QC, Canada; ^9^Advanced Baby Imaging Lab, Brown University, Providence, RI, United States; ^10^Sherbrooke Connectivity Imaging Laboratory (SCIL), Université de Sherbrooke, Sherbrooke, QC, Canada; ^11^School of Physical and Occupational Therapy, McGill University, Montreal, QC, Canada

**Keywords:** congenital heart disease, preterm, white matter microstructure, neurite orientation dispersion and density imaging, multicomponent driven equilibrium single pulse observation of T_1_ and T_2_

## Abstract

**Introduction:**

Alterations to white matter microstructure as detected by diffusion tensor imaging have been documented in both individuals born with congenital heart disease (CHD) and individuals born preterm. However, it remains unclear if these disturbances are the consequence of similar underlying microstructural disruptions. This study used multicomponent driven equilibrium single pulse observation of T_1_ and T_2_ (mcDESPOT) and neurite orientation dispersion and density imaging (NODDI) to characterize and compare alterations to three specific microstructural elements of white matter – myelination, axon density, and axon orientation – in youth born with CHD or born preterm.

**Methods:**

Participants aged 16 to 26 years with operated CHD or born ≤33 weeks gestational age and a group of healthy peers of the same age underwent a brain MRI including mcDESPOT and high angular resolution diffusion imaging acquisitions. Using tractometry, average values of myelin water fraction (MWF), neurite density index (NDI), and orientation dispersion index (ODI) were first calculated and compared between groups for 30 white matter bundles. Afterwards, bundle profiling was performed to further characterize the topology of the detected microstructural alterations.

**Results:**

The CHD and preterm groups both presented with widespread bundles and bundle segments with lower MWF, accompanied by some occurrences of lower NDI, relative to controls. While there were no differences in ODI between the CHD and control groups, the preterm group presented with both higher and lower ODI compared to the control group and lower ODI compared to the CHD group.

**Discussion:**

While youth born with CHD or born preterm both presented with apparent deficits in white matter myelination and axon density, youth born preterm presented with a unique profile of altered axonal organization. Future longitudinal studies should aim to better understand the emergence of these common and distinct microstructural alterations, which could orient the development of novel therapeutic approaches.

## Introduction

Individuals born with congenital heart disease (CHD) or born preterm both commonly present with neonatal acquired brain injury (1, 2), brain dysmaturation (3, 4), and similar profiles of neurodevelopmental difficulties across the lifespan (5–7). Neonatal brain injuries in both groups include a characteristic pattern of diffuse white matter injury (WMI) (8, 9). Studies of animal models of CHD and prematurity have demonstrated that these diffuse WMIs are associated with the selective death and arrested maturation of pre-myelinating late oligodendrocyte progenitors (preOLs) (10, 11). These progenitor cells are particularly vulnerable to insults such as hypoxia-ischemia and inflammation (12), which are conditions frequently encountered by both neonates with CHD and neonates born preterm during their perinatal course and are believed to lead to WMI (8, 13).

Due to the critical role of mature oligodendrocytes in myelinating and providing trophic support to axons (14), this interruption to the oligodendrocyte lineage is hypothesized to have a persistent impact on white matter maturation in survivors of CHD or preterm birth across the lifespan. Consistent with this hypothesis, previous diffusion tensor imaging (DTI) studies have demonstrated alterations to white matter microstructure in adolescents and young adults born with CHD (15–21) or born preterm (22–29). However, due to the relatively non-specific nature of DTI metrics, which can reflect numerous microstructural elements of white matter (30), it remains unclear if these DTI findings are driven by the same underlying microarchitectural alterations in these two populations.

Recently developed quantitative magnetic resonance imaging (MRI) techniques can provide more specific insight into the nature of these previously reported white matter microstructural alterations. Of these, multicomponent driven equilibrium single pulse observation of T_1_ and T_2_ (mcDESPOT) allows for the calculation of the myelin water fraction (MWF), a more specific marker of myelin content (31). In addition, neurite orientation dispersion and density imaging (NODDI) allows for the calculation of the neurite density index (NDI) and the orientation dispersion index (ODI), indices of apparent axon density and orientation dispersion, respectively, in white matter (32). Our group was the first, and to our knowledge, the only to apply mcDESPOT in the CHD population to detect widespread lower white matter myelination in youth born with CHD as compared to controls (33). This technique has yet to be applied to the preterm population. We also previously used NODDI in youth born with CHD and observed lower apparent axon density in the corpus callosum and many association bundles, with relatively preserved axon orientation, as compared to controls (17). Others have also used NODDI to detect lower apparent axon density and altered axon orientation dispersion in the white matter of neonates with CHD (34) and young children born preterm (35–38). However, there is currently no direct, quantitative comparison of white matter microstructure between these two populations. Therefore, it remains unclear how long-lasting white matter dysmaturation may differ in microstructural specificity or in topology between individuals born with CHD and individuals born preterm.

As such, the objective of this study was to assess and compare three specific elements of white matter microstructure – myelination, axon density, and axon orientation – between youth born with CHD, youth born preterm, and healthy peers. To accomplish this, we employed tractometry to characterize MWF, NDI, and ODI in 30 white matter bundles. We first compared bundle-average values of these metrics between the three groups. We then explored if bundle profiling of the affected white matter bundles could more precisely characterize the topology of the detected microstructural alterations.

## Materials and methods

### Participants

The study sample included three groups of participants aged 16 to 26 years old: (i) individuals born with CHD, (ii) individuals born preterm, and (iii) healthy controls. The CHD group was comprised of term-born individuals with complex CHD who underwent open-heart surgery using cardiopulmonary bypass before two years of age. We first recruited participants for the CHD group from lists of eligible participants who completed a previous research study at the McGill University Health Centre (39). Additional participants were enrolled directly from the cardiology clinics of the Montreal Children’s Hospital and Royal Victoria Hospital.

The preterm group was comprised of individuals born preterm at ≤33 weeks of gestational age without complex CHD. We recruited participants for the preterm group from lists of eligible participants from two previously-completed studies, the first at the McGill University Health Centre (39) and the second at the Centre Hospitalier Universitaire Sainte-Justine (40). Additional preterm participants were recruited from the neonatal follow-up programs of the Montreal Children’s Hospital and the Jewish General Hospital.

Term-born, typically-developing individuals who did not have a history of any neurological or developmental conditions and had not previously received rehabilitation or special education services during childhood or adolescence formed our control group. Participants in the control group were recruited from the community and local educational institutions through physical and online advertisements and word of mouth.

Exclusion criteria for all groups included documented genetic or chromosomal abnormalities, cerebral palsy, prior history of brain tumor or malformation, documented traumatic brain injury, multi-organ dysmorphic conditions, congenital infection, contraindications for MRI, and inability to communicate in English or French. Written informed consent was provided by participants aged 18 years and older and by the legal guardians of participants younger than 18 years of age. Participants younger than 18 years of age provided written informed assent. This study was approved by the Pediatric Research Ethics Board of the McGill University Health Centre.

### MRI acquisition

All participants completed a brain MRI at the Montreal Children’s Hospital on a 3 Tesla MRI system (Achieva X, Philips Healthcare, Best, The Netherlands) using a 32-channel head coil. The MRI protocol included T1-weighted, mcDESPOT, and high angular resolution diffusion imaging (HARDI) acquisitions. The T1-weighted image was acquired with a turbo field echo pulse sequence (TR = 8.1 ms, TE = 3.7 ms, TI = 1,010 ms, flip angle = 8°, voxel size = 1.00 × 1.00 × 1.00 mm^3^). All T1-weighted images were reviewed by an experienced neuroradiologist, blinded to the participants’ medical histories, who identified overt brain abnormalities and classified them based on their probable origin as either acquired or developmental. The mcDESPOT acquisition included a series of ten spoiled gradient recalled echo (SPGR) sequences (TR = 6.7 ms, TE = 3.7 ms, flip angle range = 2° to 18°, voxel size = 1.67 × 1.67 × 1.70 mm^3^), eight balanced steady-state free precession (bSSFP) sequences (TR = 6.8 ms, TE = 3.4 ms, flip angle range = 12° to 70°, voxel size = 1.67 × 1.67 × 1.70 mm^3^) acquired at 0° and 180° phase-cycling, and an inversion recovery SPGR (IR-SPGR) sequence (TR = 6.5 ms, TE = 3.2 ms, TI = 450 ms, flip angle = 5°, voxel size = 1.67 × 1.67 × 1.70 mm^3^). The HARDI acquisition (TR = 9,400 ms, TE = 78 ms, flip angle = 90°, voxel size = 2.00 × 2.04 × 2.00 mm^3^) included two single-shell HARDI sequences (b = 700 s/mm^2^ and 30 directions; b = 2,000 s/mm^2^ and 60 directions), each with a non-diffusion-weighted volume, as well as a non-diffusion-weighted sequence with reversed phase encoding.

### Image processing

The TractoFlow pipeline (41–43) was used to pre-process T1-weighted and diffusion-weighted data and perform DTI modeling and probabilistic particle filter tractography, as previously described (17). The outputs of the TractoFlow pipeline included DTI metric maps, a pre-processed T1-weighted image, and a whole-brain tractogram, all in diffusion space, for each participant. Afterwards, a modified version of RecoBundles (44) was used to extract 30 cerebral white matter bundles, which included bilateral association and projection bundles and eight anterior-to-posterior subdivisions of the corpus callosum. The *association bundles* included the bilateral arcuate fasciculus, cingulum, inferior frontal occipital fasciculus, inferior longitudinal fasciculus, uncinate fasciculus, and superior longitudinal fasciculus I, II, and III. The *projection bundles* included the bilateral corona radiata, corticospinal tract, and optic radiation. The *corpus callosum subdivisions* included the rostrum, anterior and posterior genu, rostral body, anterior and posterior mid-body, isthmus, and splenium.

Data from the SPGR, IR-SPGR, and bSSFP sequences were used for mcDESPOT modeling, producing three-dimensional maps of MWF for each participant as previously described (31). Each participant’s final MWF map was subsequently registered to their diffusion-space T1-weighted image and fractional anisotropy (FA) map with rigid, affine, and symmetric normalization transforms using ANTs (45), in order to ensure proper alignment of MWF maps with extracted white matter bundles.

Concurrently, pre-processed diffusion-weighted data were used for NODDI modeling with Accelerated Microstructure Imaging *via* Complex Optimization (46), producing three-dimensional maps of NDI and ODI for each participant. For the NODDI model, we used custom estimates of parallel and isotropic diffusivity priors calculated specifically for our dataset. Using a quantitative approach described previously (17), we computed a parallel diffusivity prior of approximately 1.64 × 10^−3^ mm^2^/s and an isotropic diffusivity prior of approximately 3.33 × 10^−3^ mm^2^/s.

All raw and pre-processed T1-weighted, diffusion-weighted, SPGR, IR-SPGR, and bSSFP images and MWF, NDI, and ODI maps underwent thorough visual inspection to identify unacceptable motion or signal artefact. Participants who failed this quality assurance step were excluded from analysis. In addition, each extracted white matter bundle was inspected to ensure robustness of extraction and anatomical accuracy. Individual bundles failing this quality inspection were also excluded from analysis.

### Tractometry

**Figure 1:**
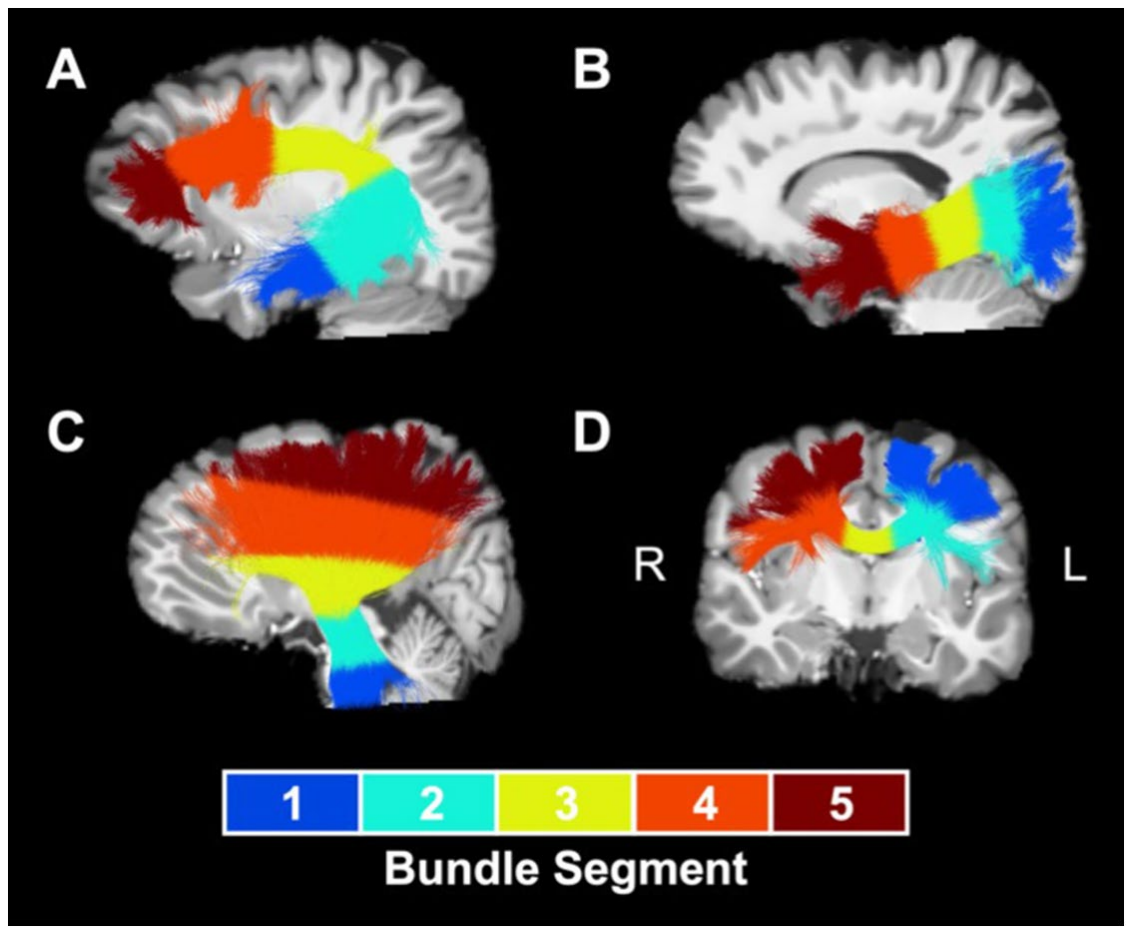
Segment numbering convention for sample bundles. Numbered segments for a representative collection of white matter bundles: **(A)** left arcuate fasciculus, **(B)** left inferior longitudinal fasciculus, **(C)** left corona radiata, and **(D)** rostral body of the corpus callosum. Bundles are overland on a T1-weighted anatomical reference image. L, left; R, right.

MWF, NDI, and ODI maps and extracted white matter bundles were used to perform tractometry (47). First, we computed bundle-average values of MWF, NDI, and ODI for each white matter bundle, calculated as the mean value of the metric within the voxels occupied by the bundle, weighted by local streamline density to reduce the impact of potentially spurious streamlines. Afterwards, in order to determine the spatial localization of group differences driving results at the whole-bundle level, we performed bundle profiling to examine MWF, NDI, and ODI values along five segments of the white matter bundles. To accomplish this, we subsampled each bundle’s centroid to five equidistant points and assigned each voxel along each bundle streamline to the closest centroid point in order to divide the bundle into five segments along its length. Segments for optic radiation and for association bundles, such as the arcuate fasciculus (Figure 1A) and the inferior longitudinal fasciculus (Figure 1B), were numbered from the more posterior bundle centroid endpoint to the more anterior endpoint. Segments for the corona radiata (Figure 1C) and the corticospinal tract were numbered from the inferior bundle centroid endpoint to the superior endpoint. Segments for corpus callosum subdivisions (Figure 1D) were numbered from the left bundle centroid endpoint to the right bundle centroid endpoint. A segment-average value of each metric was calculated for each bundle segment as the mean value of the metric within the voxels occupied by the segment, weighted by local streamline density.

### Statistical analysis

Descriptive statistics were first used to characterize the groups in terms of their individual and clinical characteristics and when possible, were compared across groups using *F*-tests for continuous variables and χ^2^ tests for categorical variables. Bundle-average and segment-average values of MWF, NDI, and ODI were compared between groups using a series of linear models, with a bundle- or segment-average metric as the dependent variable, group as the independent variable, and age and sex as covariates. These models were first fit with the control group as the reference level for the group variable to test for significant differences in metrics between the CHD group and the control group and between the preterm group and the control group. Afterwards, the models were re-fit with the CHD group as the reference level to test for significant differences between the CHD and preterm groups. In addition, bundle-average metrics were compared between participants with and without overt brain abnormalities, separately in each group, using two-sample *t*-tests. The false discovery rate correction was used to correct for multiple comparisons across the examined bundles or segments, setting the threshold of statistical significance to *q* < 0.05. Between-group comparisons of segment-average metrics were restricted to bundles showing at least one significant group difference at the bundle-average level for a given metric.

## Results

### Participant characteristics

A total of 53 youth born with CHD, 55 youth born preterm, and 56 control participants were enrolled in this study. Of these, one individual in each of the three groups was excluded from analyses due to incomplete acquisition of one of the key sequences. Additionally, eight CHD participants, 10 preterm participants, and eight controls were also excluded after failing image quality assessment due to unacceptable motion or signal artefacts. The excluded participants did not differ from the included participants in terms of age, sex, body mass index, or presence of overt brain abnormality, and the proportion of excluded participants did not differ between the three groups.

The individual characteristics of the final study sample of 44 youth born with CHD, 44 youth born preterm, and 47 controls are outlined in Table 1. Among the participants in the CHD group, 37 were born with a two-ventricle cardiac physiology (dextro-transposition of the great arteries: *n* = 15; tetralogy of Fallot: *n* = 12; ventricular septal defect: *n* = 4; double outlet right ventricle: *n* = 2; total anomalous pulmonary venous connection: *n* = 2; Ebstein’s anomaly: *n* = 1; truncus arteriosus type I: *n* = 1), while seven were born with a single-ventricle cardiac physiology (pulmonary atresia with intact ventricular septum: *n* = 3; double inlet left ventricle: *n* = 2; hypoplastic left heart syndrome: *n* = 1; tricuspid atresia: *n* = 1). Participants in the preterm group were born at a mean gestational age of 28.1 weeks (standard deviation = 2.2 weeks) with a mean birth weight of 970 g (standard deviation = 268 g).

**Table 1:** Participants’ individual characteristics.

	CHD (*N* = 44)	Preterm (*N* = 44)	Control (*N* = 47)	*p* value
**Age at MRI (years)**	19.9 ± 2.3	20.2 ± 3.1	20.7 ± 2.5	0.368
**Sex**				
Female	25 (56.8%)	24 (54.5%)	29 (61.7%)	0.778
Male	19 (43.2%)	20 (45.5%)	18 (38.3%)	
**BMI**	23.0 ± 4.6	22.3 ± 3.5	23.6 ± 3.3	0.285

Data are presented as mean ± SD for continuous variables and *n* (%) for categorical variables. BMI, body mass index; MRI, magnetic resonance imaging.

Overt brain abnormalities were detected in 10 CHD participants (22.7%), 10 preterm participants (22.7%), and five control participants (10.6%). The prevalence of overt brain abnormalities did not differ significantly between groups (*p* = 0.226). Details of these overt brain abnormalities are outlined in Table 2. Brain abnormalities likely from an acquired origin were found in seven CHD participants (15.9%), nine preterm participants (20.4%), and four control participants (8.5%). Brain abnormalities likely from a developmental origin were observed in five CHD participants (11.3%), one preterm participant (2.3%), and two control participants (4.3%).

**Table 2:** Overt brain abnormalities.

	CHD (*N* = 44)	Preterm (*N* = 44)	Control (*N* = 47)
**Overt brain abnormalities**	**10 (22.7%)**	**10 (22.7%)**	**5 (10.6%)**
**Abnormalities likely acquired in origin**	**7 (15.9%)**	**9 (10.4%)**	**4 (8.5%)**
Asymmetrical ventricles	2 (4.5%)	1 (2.3%)	1 (2.1%)
Cerebellar space occupying lesion	1 (2.3%)	-	-
Enlarged perivascular spaces	2 (4.5%)	-	-
Evidence of white matter volume loss^a^	-	7 (15.9%)	-
Evidence of grey matter volume loss^b^	-	2 (4.5%)	-
Noticeable sequelae of periventricular white matter injury	1 (2.3%)	-	-
Susceptibility signal abnormality (diffuse)	-	-	3 (6.4%)
Susceptibility signal abnormality (focal)	2 (4.5%)	-	-
**Abnormalities likely developmental in origin**	**5 (11.3%)**	**1 (2.3%)**	**2 (4.3%)**
Cerebellar hypoplasia	-	1 (2.3%)	-
Chiari I malformation	1 (2.3%)	-	-
Cortical developmental anomaly	1 (2.3%)	-	-
Developmental venous anomaly	1 (2.3%)	-	2 (4.3%)
Grey matter heterotopia	3 (6.8%)	-	1 (2.1%)

a Widened ventricles and/or increased extra-axial spaces.

b Widened sulci.

### Group comparisons of MWF

**Figure 2:**
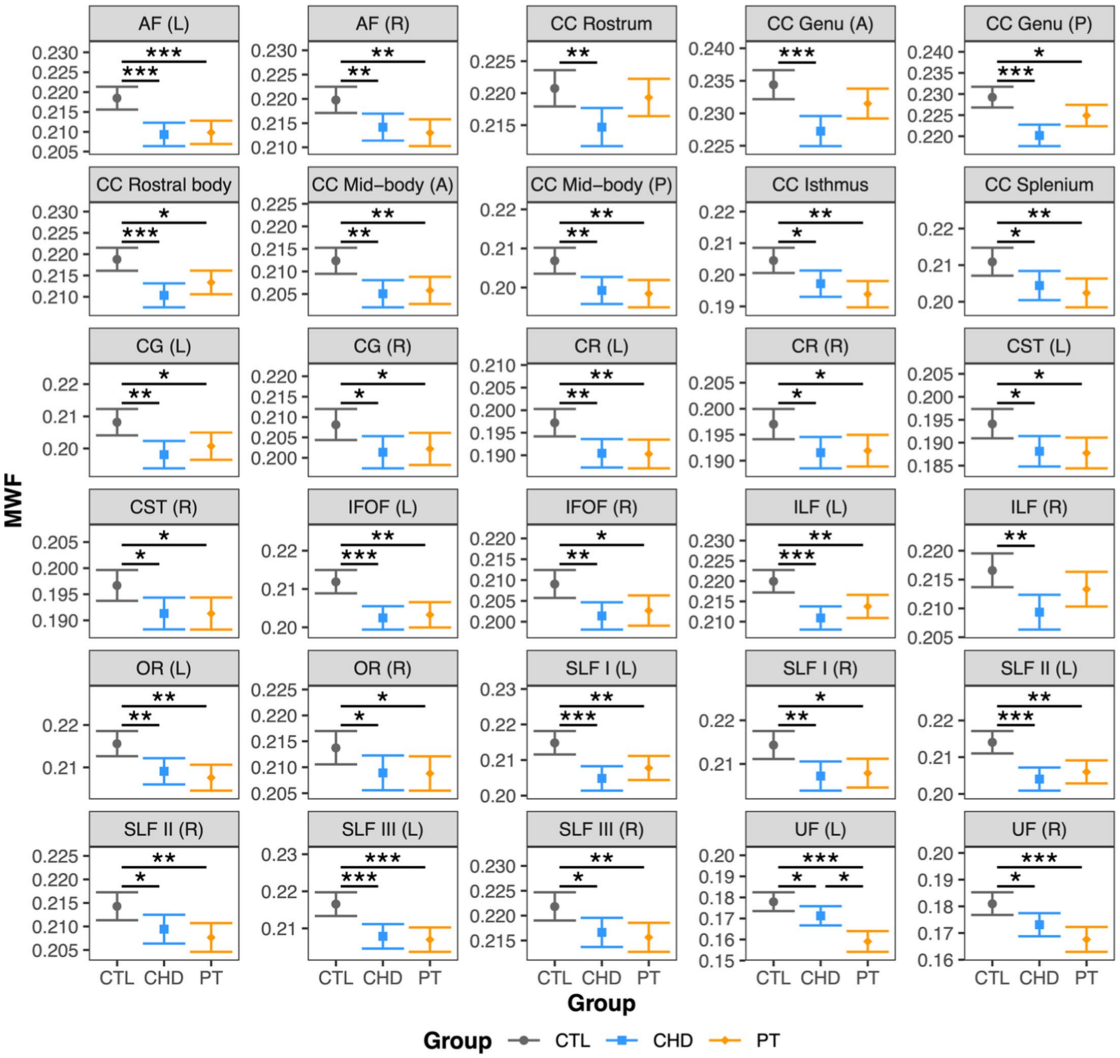
Group comparisons of bundle-average MWF. Data are presented as age- and sex-adjusted mean [95% confidence interval]. */**/*** = q < 0.05/0.01/0.001. A, anterior; AF, arcuate fasciculus; CC, corpus callosum; CG, cingulum; CHD, congenital heart disease (blue); CR, corona radiata; CST, corticospinal tract; CTL, control (grey); IFOF, inferior frontal occipital fasciculus; ILF, inferior longitudinal fasciculus; L, left; OR, optic radiation; P, posterior; PT, preterm (orange); SLF, superior longitudinal fasciculus; R, right; UF, uncinate fasciculus.

At least one group difference in bundle-average MWF was observed in all 30 white matter bundles (Figure 2). As compared to the control group, MWF was significantly lower in the CHD group in all 30 bundles and in the preterm group in 27 bundles, representing all bundles except the right inferior longitudinal fasciculus and the rostrum and anterior genu of the corpus callosum. Bundle-average MWF was only significantly different between the CHD and preterm groups in the left uncinate fasciculus, whereby lower MWF relative to controls was more pronounced in the preterm group than the CHD group. Bundle-average MWF did not differ between participants with and without overt brain abnormalities in any group.

**Figure 3:**
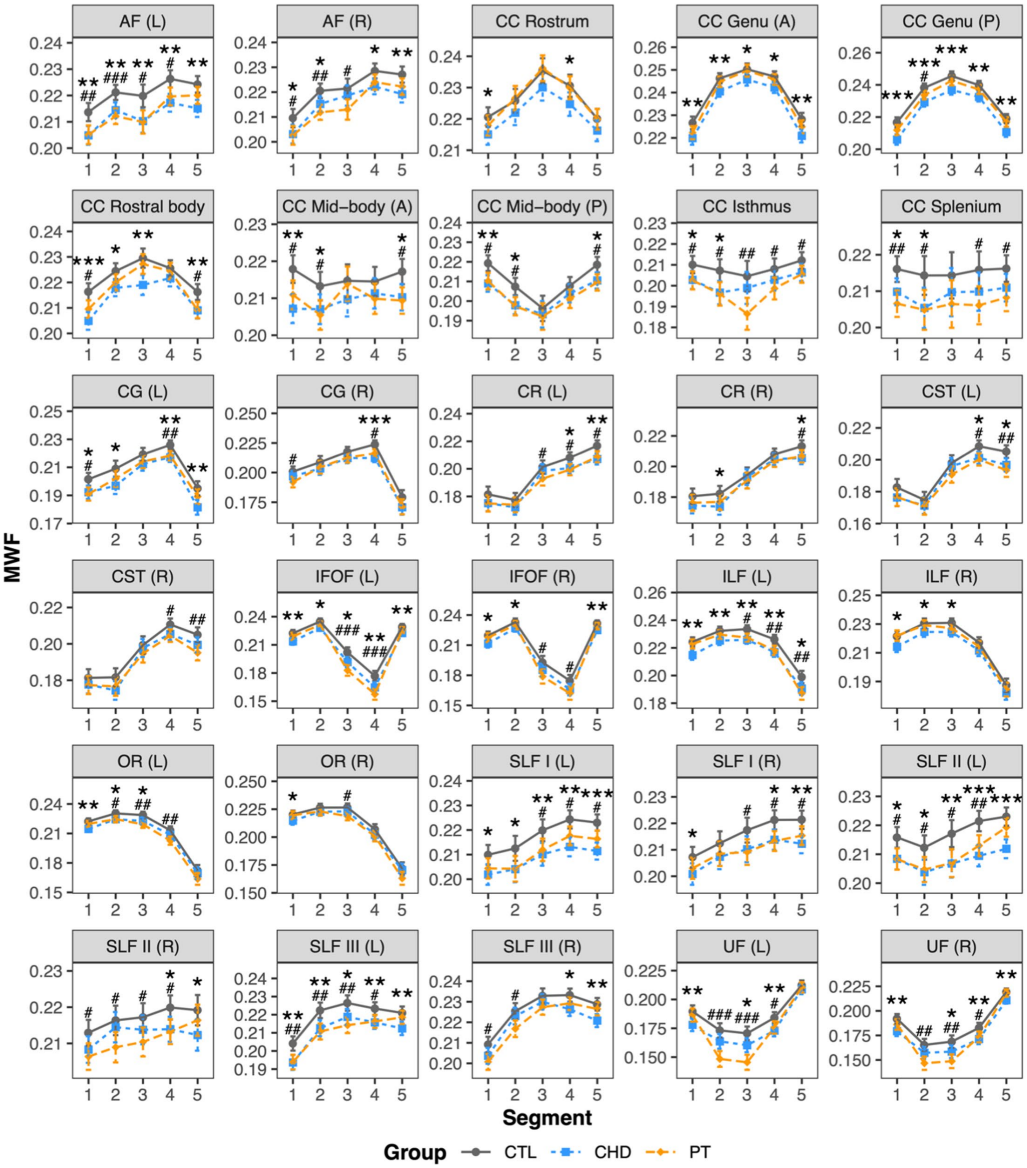
Group comparisons of bundle MWF profiles. Data are presented as age- and sex-adjusted mean [95% confidence interval]. */**/*** = q < 0.05/0.01/0.001 for CHD vs. CTL; #/##/### = q < 0.05/0.01/0.001 for PT vs. CTL. A,  anterior; AF ,  arcuate fasciculus; CC ,  corpus callosum; CG ,  cingulum; CHD,  congenital heart disease (blue); CR ,  corona radiata, CST,  corticospinal tract; CTL ,  control (grey); IFOF ,  inferior frontal occipital fasciculus; ILF ,  inferior longitudinal fasciculus; L ,  left; OR ,  optic radiation; P,  posterior; PT,  preterm (orange); SLF ,  superior longitudinal fasciculus; R ,  right; UF,  uncinate fasciculus.

Bundle profiling of MWF revealed many bundle segments with lower MWF in the CHD and preterm groups relative to controls, with no differences in segment-average MWF between the CHD and preterm groups (Figure 3). In the association bundles, findings of lower MWF relative to controls in the CHD and preterm groups were widespread and affected a combination of anterior, central, and posterior segments, with no unifying pattern across bundles in the spatial localization of lower MWF in either group. In the projection bundles, significantly lower MWF relative to controls in both clinical groups tended to be more frequent in the superior segments of the corona radiata and corticospinal tract (segments #4 and #5) and the posterior segments of the optic radiation (segments #1 to #3), although some differences were observed in other segments. In the corpus callosum, observations of lower MWF relative to controls in both clinical groups were more frequent in the fanning cortical segments (segments #1, #2, #4, and #5) of the corpus callosum subdivisions than in the central commissural segment (segment #3).

### Group comparisons of NDI

**Figure 4:**
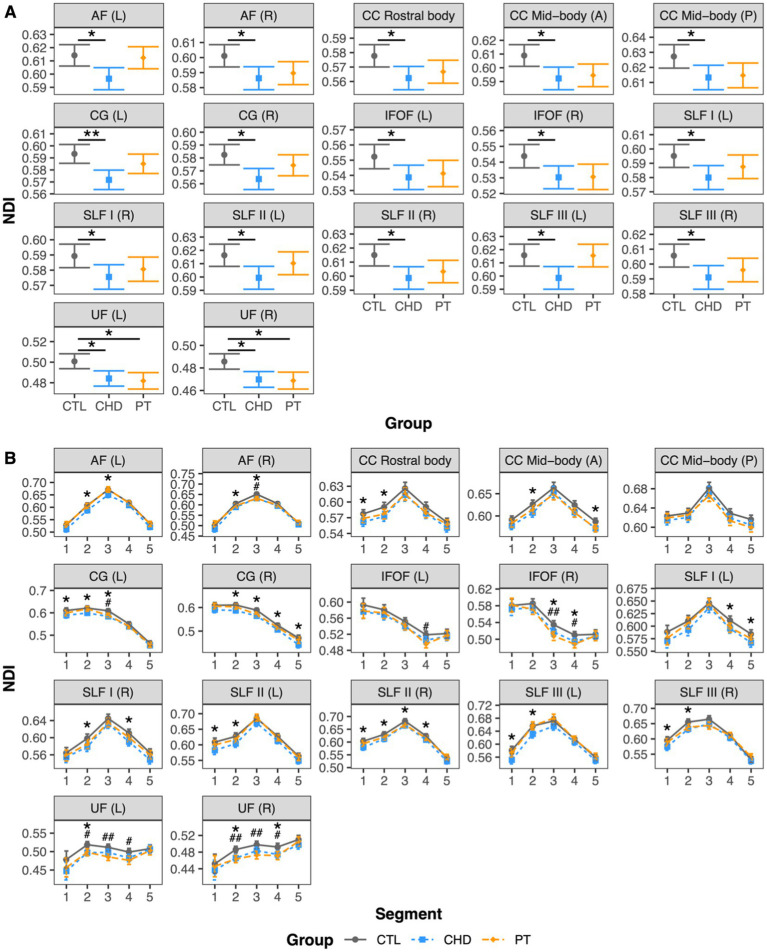
Group comparisons of NDI. Data are presented as age- and sex-adjusted mean [95% confidence interval]. **(A)** Group comparisons of bundle-average NDI. */**/*** = q < 0.05/0.01/0.001. **(B)** Group comparisons of bundle NDI profiles. */**/*** = q < 0.05/0.01/0.001 for CHD vs. CTL; #/##/### = q < 0.05/0.01/0.001 for PT vs. CTL. A, anterior; AF, arcuate fasciculus; CC, corpus callosum; CG, cingulum; CHD, congenital heart disease (blue); CTL, control (grey); IFOF, inferior frontal occipital fasciculus; L, left; P, posterior; PT, preterm (orange); SLF, superior longitudinal fasciculus; R, right; UF, uncinate fasciculus.

At least one group difference in bundle-average NDI was observed in 17 white matter bundles, including many bilateral association bundles and central subdivisions of the corpus callosum (Figure 4A). NDI was lower in the CHD group as compared to controls in all 17 of these bundles. NDI was lower in the preterm group as compared to controls in only two bundles, namely the left and right uncinate fasciculus, although non-significant trends toward lower NDI in the preterm group when compared to controls were observed in many other bundles. There were no significant differences in bundle-average NDI between the CHD and preterm groups, or between participants with and without overt brain abnormalities in any group.

Bundle profiling of NDI in these 17 bundles (Figure 4B) revealed that segment-average NDI was lower in the CHD group as compared to controls in segments across 15 bundles. In the association bundles, there was no clear pattern in the spatial localization of lower NDI along the length of the bundles. In the corpus callosum subdivisions, lower NDI in the CHD group occurred in the fanning cortical segments (segments #1, #2, and #5). Of note, in the posterior mid-body of the corpus callosum and the left inferior frontal occipital fasciculus, there were no differences in segment-average NDI between the CHD and control groups despite findings at the whole-bundle level, with only non-significant trends toward lower NDI in the CHD group along the length of the bundles. With respect to the preterm group, lower NDI at the bundle-average level was driven by alterations in the curved segments (segments #2, #3, and #4) of the bilateral uncinate fasciculus. We also observed isolated segments of lower NDI in the preterm group compared to controls in several other association bundles, reflecting minor differences that were not pronounced enough to manifest as significant differences at the whole-bundle level. Finally, there were no differences in segment-average NDI between the CHD and preterm groups.

### Group comparisons of ODI

**Figure 5:**
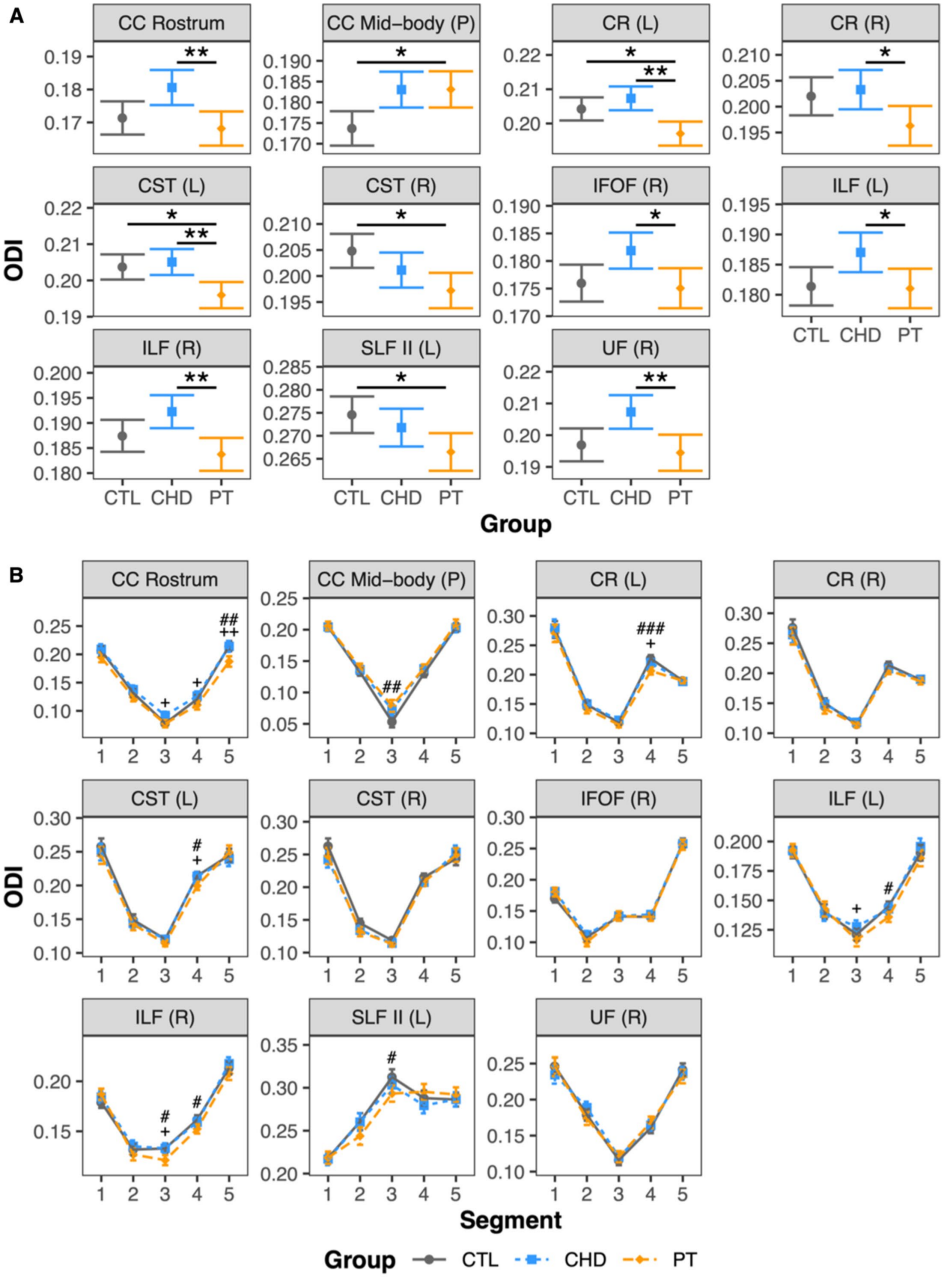
Group comparisons of ODI. Data are presented as age- and sex-adjusted mean [95% confidence interval]. **(A)** Group comparisons of bundle-average ODI. */**/*** = q < 0.05/0.01/0.001. **(B)** Group comparisons of bundle ODI profiles. #/##/### = q < 0.05/0.01/0.001 for PT vs. CTL; +/++/+++ = q < 0.05/0.01/0.001 for PT vs. CHD. CC, corpus callosum; CHD, congenital heart disease (blue); CR, corona radiata; CST, corticospinal tract; CTL, control (grey); IFOF, inferior frontal occipital fasciculus; ILF, inferior longitudinal fasciculus; L, left; P, posterior; PT, preterm (orange); SLF, superior longitudinal fasciculus; R, right; UF, uncinate fasciculus.

At least one group difference in bundle-average ODI was observed in 11 white matter bundles including a mixture of corpus callosum subdivisions and projection and association bundles (Figure 5A). There were no differences in bundle-average ODI between the CHD and control groups. The preterm group presented with differences in bundle-average ODI relative to the control group in five bundles, with higher ODI in the posterior mid-body of the corpus callosum and lower ODI in the left corona radiata, bilateral corticospinal tract, and left superior longitudinal fasciculus II. When compared to the CHD group, bundle-average ODI was lower in the preterm group in eight bundles. Only two of these eight bundles overlapped with bundles in which lower ODI was observed in the preterm group compared to controls. In the other six bundles, differences between the preterm and control groups were driven by non-significant trends toward lower ODI in the preterm group and/or higher ODI in the CHD group relative to controls. Bundle-average ODI did not differ between participants with and without overt brain abnormalities in any group.

**Figure 6:**
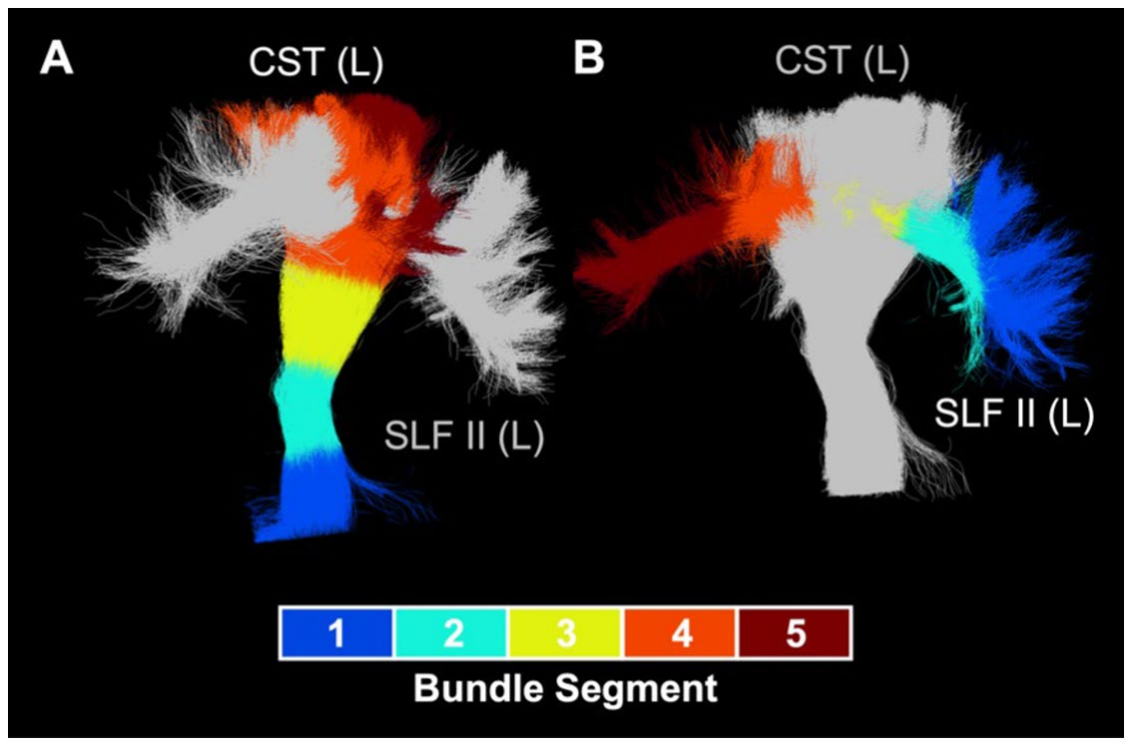
Intersection of left corticospinal tract and left superior longitudinal fasciculus II. **(A)** Visualization of intersection between left superior longitudinal fasciculus II (silver) and left corticospinal tract segment #4 (orange). **(B)** Visualization of intersection between left corticospinal tract (silver) and left superior longitudinal fasciculus II segment #3 (yellow). CST, cortisospinal tract; L,  left; SLF,  superior longitudinal fasciculus.

Bundle profiling of ODI in these 11 bundles (Figure 5B) revealed that there were no significant differences in segment-average ODI between the CHD and control groups, consistent with our findings at the bundle-average level. When considering differences between the preterm and control groups, bundle profiling revealed that higher bundle-average ODI in the posterior mid-body of the corpus callosum in the preterm group was driven by higher segment-average ODI in the bundle’s low-ODI central commissural segment (segment #3). Lower bundle-average ODI in the preterm group relative to controls in the left corona radiata, left corticospinal tract, and left superior longitudinal fasciculus II was driven by a single segment, of relatively high ODI, in each of these bundles: segment #4 of the left corona radiata and corticospinal tract and segment #3 of the left superior longitudinal fasciculus II. Segment #4 of the left corona radiata and corticospinal tract are located at the level of centrum semiovale, while segment #3 of the left superior longitudinal fasciculus II corresponds to the intersection of this bundle with segment #4 of the corticospinal tract in the left centrum semiovale (Figure 6). When considering differences between the preterm and CHD groups, many segments with significant differences corresponded to segments where ODI was significantly lower in the preterm group compared to controls, including those outlined above, as well as a small number of isolated segments from other bundles. Other segments with significant differences in ODI between the preterm and CHD groups displayed no differences between these groups and the control group, reflecting a non-significant decrease in ODI in the preterm group and/or a non-significant increase in ODI in the CHD group relative to controls.

**Figure 7:**
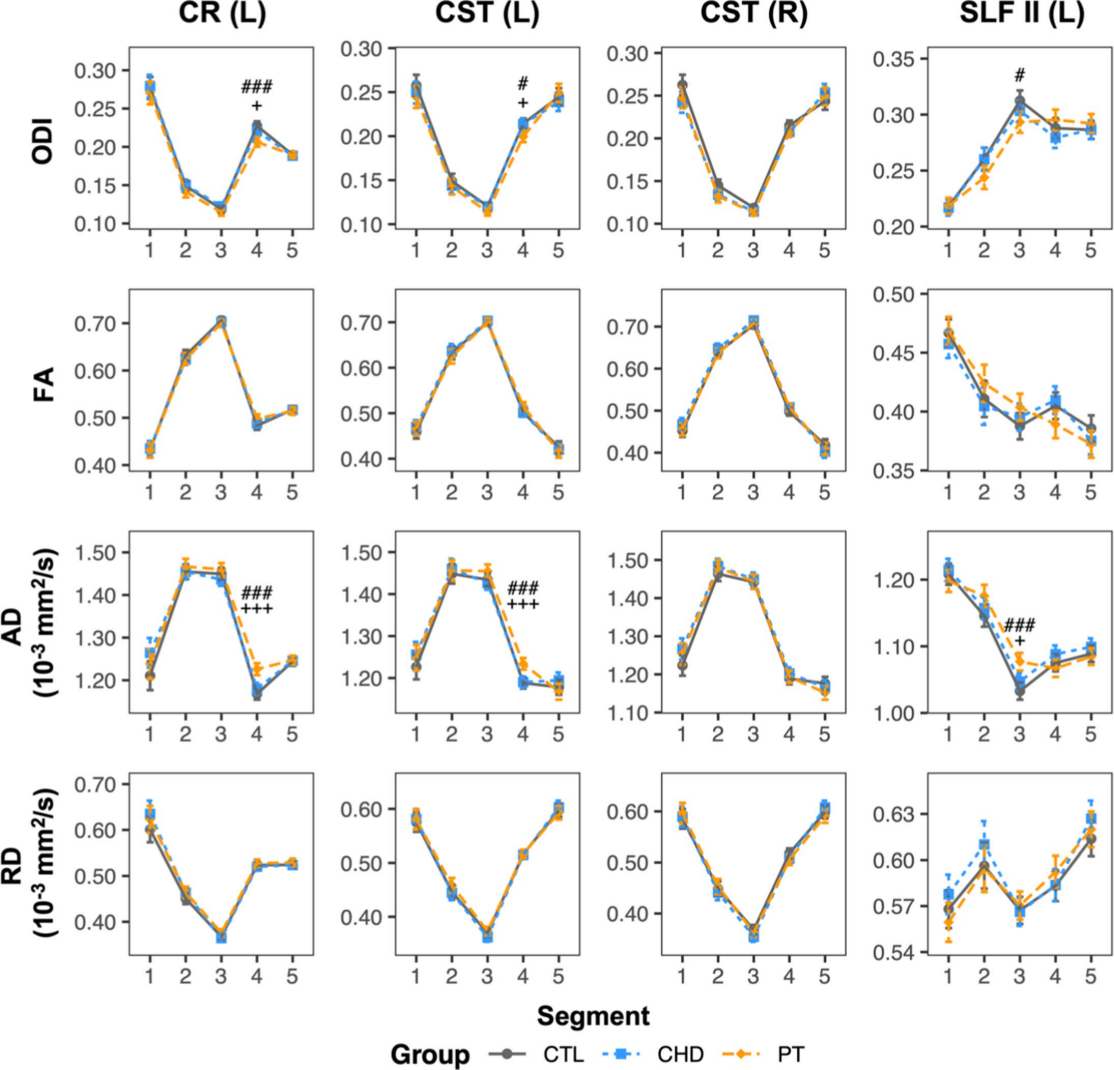
ODI and DTI metric profiles for bundles where the preterm group displayed lower bundle-average ODI relative to controls. Data are presented as age- and sex-adjusted mean [95% confidence interval]. #/##/### = q < 0.05/0.01/0.001 for PT vs. CTL; +/++/+++ = q < 0.05/0.01/0.001 for PT vs. CHD. CHD, congenital heart disease (blue); CR, corona radiata; CST, corticospinal tract; CTL, control (grey); L, left; PT, preterm (orange); SLF, superior longitudinal fasciculus; R, right.

To facilitate a better understanding of the microstructural alterations underlying the unique observations of lower bundle-average ODI in the preterm group relative to controls, bundle profiling of FA, axial diffusivity (AD), and radial diffusivity (RD) for the four affected bundles was performed (Figure 7). These DTI profiles revealed that lower ODI in segment #4 of the left corona radiata and corticospinal tract and in segment #3 of the left superior longitudinal fasciculus II was accompanied by significantly higher AD in these same segments. No other differences were observed.

### Summary of group comparisons

A summary of the key findings of the comparisons of MWF, NDI, and ODI among the CHD, preterm, and control groups is provided in Table 3.

**Table 3:** Summary of group comparisons of MWF, NDI, and ODI.

	CHD vs. Control	Preterm vs. Control	Preterm vs. CHD
MWF	Widespread ↓ MWF.	Widespread ↓ MWF.	↓ MWF in the left uncinate fasciculus only.
NDI	↓ NDI in association bundles and corpus callosum subdivisions.	↓ NDI in bilateral uncinate fasciculus and other isolated bundle segments.	No significant differences.
ODI	No significant differences.	↑ ODI in single-fiber region of corpus callosum. ↓ ODI, mostly in crossing-fiber regions.	↓ ODI in isolated segments of several bundles.

## Discussion

This study provides the first specific comparison of white matter microstructure alterations between individuals born with CHD or born preterm, using two complementary advanced quantitative MRI techniques, mcDESPOT and NODDI. In doing so, we demonstrate that youth born with CHD or born preterm display a similar pattern of widespread myelination deficits, likely linked to their common pattern of neonatal WMI. Both groups also displayed lower apparent axon density as compared to controls, although these differences were more widespread in the CHD group. Interestingly, the two groups were distinct from one another in terms of axon orientation dispersion, wherein significant differences in ODI relative to controls were limited to the preterm group and the preterm group had lower ODI than the CHD group in several white matter bundles. Our findings highlight that despite both presenting with diffuse WMI during infancy, youth born with CHD or born preterm differ to some extent with respect to the specific microstructural elements underlying their long-term white matter dysmaturation.

### Myelination deficits in youth born with CHD or born preterm

Youth born with CHD or born preterm both presented with lower bundle- and segment-average MWF in many bundles as compared to controls, occurring in a widespread fashion across the white matter and impacting association, projection, and commissural bundles alike. Additionally, there were very limited differences in MWF between the CHD and preterm groups, with only one difference at the bundle-average level, driven by more pronounced MWF reductions in the preterm group in the left uncinate fasciculus, and no differences at the bundle-segment level. Our findings suggest that deficient myelination is a long-lasting white matter alteration common to both clinical groups, which is consistent with our understanding of the primary cellular target of their frequent neonatal diffuse WMI, the preOL. As compared to neurons, other glial cells, and more mature stages of the oligodendrocyte lineage, preOLs have a heightened vulnerability to hypoxic–ischemic and inflammatory insults (12). Accordingly, diffuse WMI in neonates with CHD or born preterm is thought to involve an acute period of selective degeneration of preOLs, with relative sparing of neurons, followed by reactive gliosis and subsequent failure of preOLs, once replenished, to differentiate into mature, myelinating stages of the oligodendrocyte lineage (10, 11). As such, long-lasting deficient myelination is an expected consequence of the pathological cascade associated with diffuse WMI in the CHD and preterm populations and is confirmed by our present observations.

Given the proposed mechanistic link of neonatal WMI and myelination failure, we expected that there may be differences in the spatial topology of alterations in MWF between our two clinical groups, in light of previous work that has established differences in the spatial topology of WMI between term-born neonates with CHD and preterm neonates (48). Indeed, it is suggested that WMI occurs most frequently in the central white matter in preterm neonates, whereas in term-born neonates with CHD, WMI tends to be more localized to the anterior and posterior white matter, with relative sparing of the central white matter (48). The authors of this study proposed that these distinct spatial lesion topologies may reflect an interplay between the earlier timing of WMI in preterm neonates as compared to term-born neonates with CHD and the earlier maturation of preOLs in central white matter regions than those in posterior and anterior regions (49). In contrast with these observations of spatially distinct patterns of neonatal WMI, we observed widespread myelination deficits, affecting anterior, central, and posterior bundles and bundle segments, in both youth with CHD and youth born preterm, with no clear differences in spatial topology of MWF alterations between the two groups. This suggests that myelination failure may be a more global white matter dysmaturational phenomenon than what has been previously described in the neonatal brain. In particular, the reactive gliosis that leads to chronic maturational failure of preOLs, involving the release of various inhibitory molecular signals from reactive astrocytes and pro-inflammatory microglia (10, 50), may extend beyond the boundaries of neonatal focal white matter lesions that can be identified on conventional MRI.

### Deficits in apparent axon density in youth born with CHD or born preterm

In addition to lower MWF, both the CHD and preterm groups presented with instances of lower NDI as compared to controls, mostly affecting association bundles, indicating reductions in apparent axon density are present in both populations during adolescence and young adulthood. While observations of lower NDI relative to controls were more frequent in the CHD group than in the preterm group, there were no differences in bundle- or segment-average NDI between the two clinical groups, indicating that axon density is not a substantially distinct microstructural element between the two groups. In addition, there were no clear differences in the distribution of NDI deficits among central versus anterior or posterior association bundle segments between the two groups, indicating that the deficits do not follow the previously reported distinct spatial patterns of neonatal WMI in either population (48). Our findings are consistent with previous findings of lower NDI relative to controls in neonates with CHD (34), as well as a prior study using a brain network-based approach that reported lower NDI across global, hub, and peripheral networks in young adults born preterm (51). However, studies in school-aged children born preterm have provided mixed evidence, reporting both lower NDI (36, 37) or no differences in NDI (35, 38) relative to controls.

It is possible that the observations of lower NDI may simply reflect deficient myelination in the two clinical groups, as reduced myelination would increase the space between axons and result in lower estimates of NDI. However, given that our observations of lower MWF relative to controls were more widespread than those of lower NDI, it seems unlikely that myelination is the only major factor influencing NDI. One possibility is that lower NDI reflects axonal loss as a downstream consequence of preOL dysmaturation, with secondary axonal loss occurring due to loss of trophic, structural, and metabolic support from mature oligodendrocytes (14, 52). Lower NDI could also reflect primary axon loss in the context of pan-cellular death within cystic white matter lesions; however, this pattern of severe brain injury is rare in contemporary cohorts of neonates born with CHD or born preterm, and is a less likely mechanism. Another possibility is that lower NDI could reflect developmental interruptions to ongoing axonal packing that occurs during typical white matter maturation through adolescence (53, 54) and young adulthood (55). Indeed, the majority of NDI alterations were localized in association bundles, which tend to mature later than projection and commissural bundles (56). The predilection for NDI deficits in later-maturing white matter bundles may suggest that these NDI alterations may not be permanent, and rather may reflect protracted maturation of these white matter regions that will eventually reach maturity. This could be confirmed by future longitudinal studies with serial characterization of NDI beyond early adulthood.

### Alterations in axon orientation dispersion specific to preterm-born youth

Interestingly, despite being linked by similar profiles of myelination and axon density alterations, the white matter maturational profiles of youth born with CHD and youth born preterm were distinguished by different patterns of axon orientation dispersion. While the CHD group presented with no alterations in ODI relative to the control group, as previously reported (17), the preterm group presented with ODI alterations in several bundles as compared to both the control and CHD groups. We observed higher ODI in the preterm group relative to controls in the posterior mid-body of the corpus callosum, which is consistent with previous reports of widespread higher ODI in school-aged children born preterm as compared to controls, including in the corpus callosum (35–38). Bundle profiling revealed that higher ODI at the bundle-average level was driven by higher ODI in the central commissural segment of this bundle. As compared to the other segments along the bundle, this segment displayed the lowest values of ODI, likely reflecting the strongly coherent organization of axons in the commissural portions of the corpus callosum (57). We can infer that higher ODI in this segment in the preterm group may reflect an enduring consequence of pathological disruptions, secondary to hypoxia-ischemic or inflammatory insults, to the coherent alignment and organization of corpus callosum axons during its ongoing maturation in the early postnatal period (58, 59). Consistent with this hypothesis, postnatal systemic inflammation has been shown to be associated with trend-level increases in ODI in the corpus callosum in newborn rats (60).

In addition, ODI was significantly lower in the preterm group in several bundles relative to controls. Importantly, the preterm group also had significantly lower ODI than the CHD group, suggesting that lower regional axon orientation dispersion is a substantially distinct neuropathology between youth born preterm and youth born with CHD. Bundle-average ODI was lower in the preterm group as compared to controls in the left corona radiata, bilateral corticospinal tract, and left superior longitudinal fasciculus II. Bundle profiling revealed these findings to be driven by lower ODI, accompanied by higher AD, in the segments in which these bundles intersect in the left centrum semiovale. These findings are consistent with one previous study of young children born preterm that reported lower ODI relative to controls in regions including the superior and posterior corona radiata and the superior longitudinal fasciculus (37), but conflicts with other studies that reported only ODI increases in preterm-born school-aged children (35, 36, 38). In addition, our findings also align with a previous report of higher AD and higher FA in the intersecting crossing-fiber regions of the corticospinal tract and superior longitudinal fasciculus in the centrum semiovale in adolescents born preterm as compared to controls (24). In line with their crossing-fiber architecture, which would involve a high degree of angular variation in axon orientation, the affected segments displayed relatively high ODI as compared to other segments along the length of the bundles in question. As such, low ODI in the preterm group in these segments may reflect a pathological loss of fiber architecture complexity. The central localization of these observations in the centrum semiovale, the preferential site of white matter lesions in preterm neonates (61, 62), may indicate a relationship to previous neonatal WMI. Indeed, one previous study reported lower ODI in preterm-born 6-year-old children with a history of neonatal WMI as compared those without a history of neonatal WMI in the right superior corona radiata, which was the region where the majority of lesions were identified in the study’s cohort (38). In addition, trend-level reductions in axon dispersion, measured both histologically and with NODDI, have been reported within demyelinating white matter lesions in spinal cord specimens from multiple sclerosis patients (63). As such, we propose that our findings of lower ODI in the preterm group may reflect an acquired loss of fiber architecture complexity within the boundaries of neonatal white matter lesions.

In the context of this proposed mechanism, it is presently unclear why lower axon dispersion was a distinguishing microstructural alteration of the preterm group only. Insight into this distinction may be gained by considering differences in the perinatal experiences of the two populations. One possibility is that the brains of preterm neonates are more vulnerable to this pattern of altered axonal architecture within white matter lesions due to the earlier developmental timing of WMI in preterm neonates. This distinction may also arise from differences in the initial insults triggering WMI in the two populations. For example, the perinatal infections commonly experienced by preterm neonates have been found to be associated with heightened risk for WMI (64, 65), while infection has been found to not be a significant risk factor for WMI in neonates with CHD (66). Therefore, the unique alterations to axonal architecture complexity observed in the preterm group could be related to distinct effects of infection/inflammation or a unique interplay between infection/inflammation and hypoxia-ischemia in preterm neonates (67). Unfortunately, due to the absence of neonatal MRI and the limited availability of neonatal clinical data for our participants, we cannot directly investigate these relationships. Future research, involving controlled experiments in animal models of preterm WMI and longitudinal studies in individuals born preterm tracking the relationship between early-life WMI, perinatal exposures such as infection/inflammation, and ongoing axonal organization are required to further explore the etiology of these observations.

### Limitations

The reported findings need to be considered in the context of a number of limitations. First, mcDESPOT and NODDI only provide a single estimate of MWF, NDI, or ODI per voxel, yet up to 90% of white matter voxels in the brain contain crossing fiber populations (68). Consequently, we were unable to determine if alterations to MWF, NDI, and ODI in intersecting bundle segments reflect a collective disruption to all present fiber populations in the region, or if they rather are the result of a selective microstructural disruption to a particular fiber population. Future studies may want to explore the added contribution of other MRI techniques that are able to characterize individual fiber populations in voxels with crossing fibers, such as fixel-based analysis (69). Lastly, although previous work has validated that MWF, NDI, and ODI are correlated with their proposed histological counterparts (70–73), these indices are indirect, proxy measures of myelination, axon density, and axon orientation. Confirmation of the apparent microstructural alterations uncovered in our study would only be achieved through post-mortem histological studies.

## Conclusion

In conclusion, this study has demonstrated that both youth born with CHD and youth born preterm present with similar profiles of widespread, deficient myelination accompanied by some occurrences of lower axon density, probably related to enduring developmental sequelae of their common early-life WMI. Despite these similarities, while youth born with CHD had relatively preserved axonal organization compared to controls, youth born preterm presented with altered axonal organization, including a unique pattern of lower axon orientation dispersion relative to both the control and CHD groups. This unique microstructural alteration may reflect differences in the perinatal experiences and exposures of the CHD and preterm populations, warranting further investigation.

## Data availability statement

The raw data supporting the conclusions of this article will be made available by the authors, without undue reservation.

## Ethics statement

The studies involving human participants were reviewed and approved by Pediatric Research Ethics Board of the McGill University Health Center. Written informed consent was provided by participants aged 18 years and older and by the legal guardians of participants younger than 18 years of age.

## Author contributions

KE: conceptualization, data curation, formal analysis, investigation, project administration, visualization, and writing – original draft. MK and CR: investigation and resources. CS-M: investigation. GG and SD: methodology and software. K-AN, TL, ÉC, and A-MN: resources. PW: supervision. MD: methodology, software, and supervision. MB-R: conceptualization, funding acquisition, investigation, methodology, project administration, resources, supervision, and writing – original draft. All authors contributed to reviewing and editing of the manuscript and approved of the final submitted version.

## Funding

This work was supported by funding from McGill University and the Research Institute of the McGill University Health Center. KE is currently supported by a Vanier Canada Graduate Scholarship from the Canadian Institutes of Health Research. MB-R was supported by a Canada Research Chair in Brain and Child Development.

## Conflict of interest

GG is an employee of Philips Healthcare Canada. MD is an employee, co-founder, and co-owner of Imeka Solutions Inc.

The remaining authors declare that the research was conducted in the absence of any commercial or financial relationships that could be construed as a potential conflict of interest.

## Publisher’s note

All claims expressed in this article are solely those of the authors and do not necessarily represent those of their affiliated organizations, or those of the publisher, the editors and the reviewers. Any product that may be evaluated in this article, or claim that may be made by its manufacturer, is not guaranteed or endorsed by the publisher.
